# Dengue Viral Myositis Complicated with Rhabdomyolysis and Superinfection of Methicillin-Resistant *Staphylococcus aureus*

**DOI:** 10.1155/2013/194205

**Published:** 2013-02-17

**Authors:** Vinothan Sunderalingam, Thirumavalavan Kanapathipillai, P. A. S. Edirisinghe, K. M. M. P. Dassanayake, I. H. G. S. Premawansa

**Affiliations:** ^1^Postgraduate Institute of Medicine, University of Colombo, Sri Lanka; ^2^Colombo North Teaching Hospital, Ragama, Sri Lanka; ^3^Faculty of Medicine, University of Kelaniya, Ragama, Sri Lanka

## Abstract

Dengue is endemic in Sri Lanka and the physician should be aware of different and unusual presentation of the illness. Rhabdomyolysis is a well-known complication following many viral and bacterial infections; however, only a few cases have been reported with dengue viral infections. Further occurrence of coinfection by dengue and bacteria such as methicillin-resistant *Staphylococcus aureus* (MRSA) has been underestimated, and few reports have been published so far. This case describes a 17-year-old boy who presented with prolonged severe myalgia, dark red urine, and a febrile illness that was diagnosed as having dengue viral myositis, dark red urine, and a febrile illness that was diagnosed as having dengue viral myositis complicated with rhabdomyolysis and superinfection of MRSA. Despite intensive care management, he died due to multiorgan failure. Autopsy and serological studies confirmed the diagnosis. This case stresses that red-coloured urine in dengue patients is not always due to haematuria, and if a patient's vital signs do not respond to appropriate fluid management in DHF, sepsis from a secondary pathogen including MRSA should be suspected.

## 1. Introduction

Dengue is endemic in Sri Lanka, and the diagnosis of dengue fever or dengue haemorrhagic fever (DHF) is not usually a challenge to the physician. However, the diagnosis of dengue fever with super infection and additional pathology is a diagnostic challenge as there can be atypical presentations, and if the diagnosis is delayed, the risk of morbidity and mortality increases. This paper describes a rare presentation of dengue fever.

## 2. Case Report

A 17-year-old boy was transferred to a tertiary centre with an acute febrile illness from a primary care facility on the 4th day of the illness. He had fever with severe myalgia for 4 days. He also had a productive cough with yellowish sputum, but there was no haemoptysis. Three days, he had watery diarrhoea, and during the latter part of his stay at primary care center, he developed oliguria and the urine became red in colour.

He denied substance abuse, self-ingestion of antipsychotic medications, or heavy manual exertion prior to the illness. He was not exposed to muddy water or paddy fields.

On examination, he was alert with an GCS of 15/15, but looked unwell and had a temperature of 38.4°C. He had generalized muscle tenderness with restricted movements due to muscle pain. He had a regular, low-volume pulse of 102 beats per minute. His supine blood pressure was 100/80 mm Hg. Chest auscultation detected a dual rhythm with no murmurs, and both lung fields were clear. Abdominal examination revealed right hypochondrial tenderness without any evidence of hepatosplenomegaly. Examination of the nervous system was unremarkable. 

Table 1 shows the serial complete blood counts, liver and renal profile of the patient. 

After an initial evaluation, a provisional diagnosis of dengue fever was made based upon the clinical picture and the background of a countrywide dengue epidemic. Leptospirosis, sepsis, and atypical pneumonia were considered as differential diagnoses. 

He was transferred to intensive care unit (ICU), for further management, which included treatment with paracetamol for fever and muscle pain. Blood cultures were drawn and empiric antibiotic therapy with intravenous cefotaxime and clarithromycin started to cover the possible pathogens.

He had reduced ionized calcium level of 8.16 mg/dL with elevated phosphate level of 5.4 mg/dL. Serum magnesium level was normal. Ultrasound examination of the abdomen was normal without evidence of ascites or pleural effusion.

He maintained normal clinical parameters apart from a sinus tachycardia of 110 bpm for the first ten hours after admission to ICU, and since then he developed severe tachycardia of 150–195 bpm with low blood pressure. Fluid management adjusted according to central venous pressure and inotropes started to maintain the blood pressure. Chest X-ray was normal. His two-dimensional echocardiogram was unremarkable, and sinus tachycardia impeded the ability to measure the ejection fraction accurately. The ultrasound scan of the abdomen was repeated on the fifth day of the illness and it was unremarkable. 

His urine full report was negative for red cells, but he had mild proteinuria and 6–8 pus cells per high-power field, and rhabdomyolysis was suspected and confirmed on laboratory analysis of serum creatine phosphokinase (CK) which revealed 60,130 U/L (verified on with a repeat analysis showing more than 90,000 U/L). The C-reactive protein was more than 96 mg/dL and the blood picture implied severe bacterial infection and sepsis. After consultation with microbiologist, antibiotics were changed to age appropriate doses of intravenous meropenem and benzyl penicillin. He maintained adequate urine output for 20 hours after admission. Since then, he developed oliguria and refractory hypotension which ultimately lead to anuria and death. 

Investigation results received after the patient's death were negative urine culture, insignificant *Leptospira* antibody titers, and a positive Dengue IgM antibody by ELISA. Blood culture revealed growth of methicillin resistant *Staphylococcus aureus* which was sensitive to ciprofloxacin, fusidic acid, gentamicin, netilmicin, and teicoplanin but it was resistant to cloxacillin and erythromycin.

**Figure 1:**
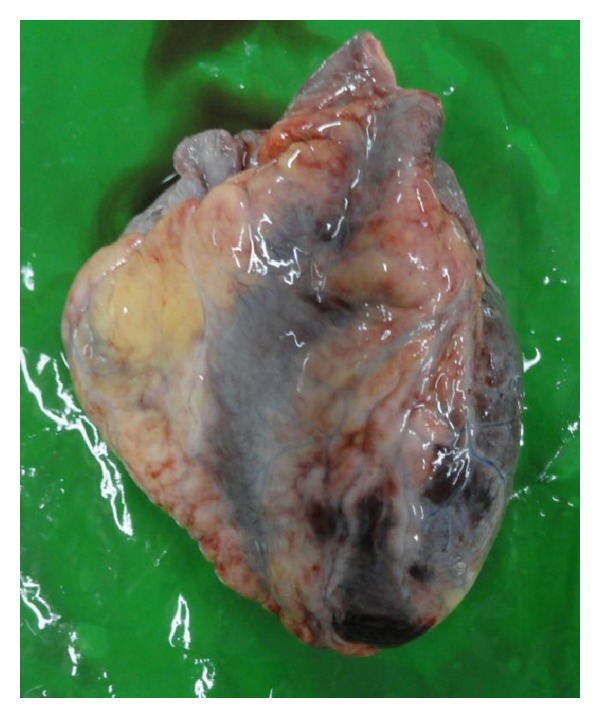
Macroscopic appearance of the heart shows multiple florid petechial to haemorrhagic areas.

**Figure 2:**
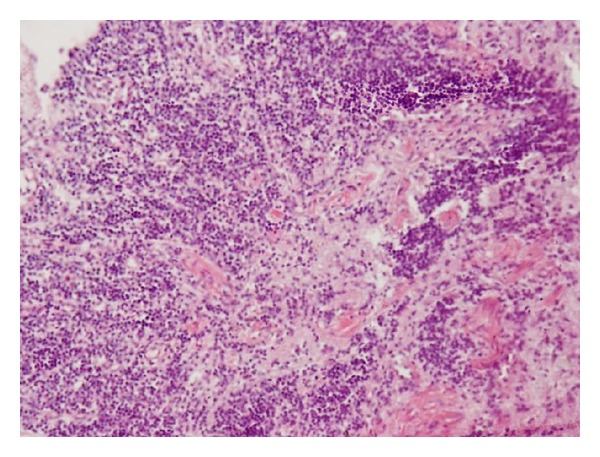
Microscopic appearance of heart shows evidence of myocarditis.

**Figure 3:**
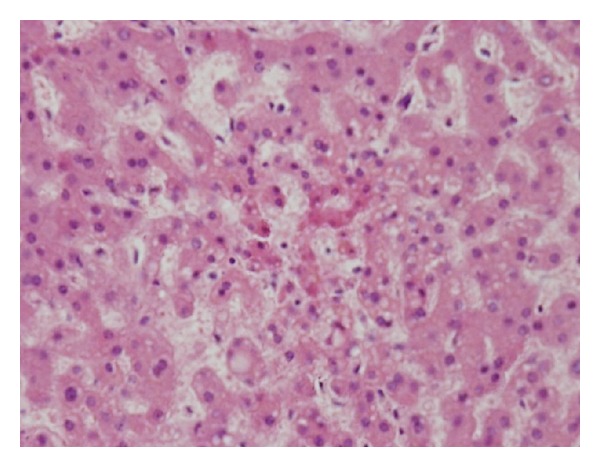
Microscopic appearance of liver shows patchy necrosis.

A medico-legal autopsy revealed tissue oedema with blood stained serous fluid in pericardial, pleural, and peritoneal cavities. The heart was flabby with multiple florid petechial to haemorrhagic areas (Figure 1). The lungs had severe oedema with an area of consolidation. The liver was enlarged with scattered areas of yellow discoloration. Histology revealed microscopic evidence of myocarditis (Figure 2), patchy necrosis of the liver (Figure 3), and renal cortices. The lungs showed bronchopneumonia. 

The cause of death was concluded as dengue viral myositis complicated with rhabdomyolysis and superinfection of MRSA leading to multiorgan failure predominantly manifesting as myocarditis, hepatic necrosis, and patchy cortical necrosis of the kidneys. 

## 3. Discussion

Rhabdomyolysis is a syndrome characterized by muscle necrosis and the release of intracellular muscle constituents into the circulation. CK levels are typically markedly elevated and muscle pain and myoglobinuria may be present. The severity of illness ranges from asymptomatic elevation of CK to life-threatening disease associated with extreme CK elevation, electrolyte imbalances, and acute kidney injury.

Viral infections leading to rhabdomyolysis are well described with several acute viral infections [1–3]. However, we have identified only 5–7 cases that reported with dengue viral myositis [1, 2]. Further dengue fever complicated with rhabdomyolysis and superinfected with MRSA has not been reported previously. Myositis and rhabdomyolysis have not been described as potential complications of dengue fever by major textbooks or review articles [1]. This is not surprising as the pathogenesis of acute viral myositis and consequent rhabdomyolysis have not been well established. Direct invasion of muscles by virus has not been consistently demonstrated, and the most likely cause is thought to be myotoxic cytokines, particularly TNF released in response to viral infection [1]. Studies of muscle biopsy specimens have revealed a range of findings, from mild lymphocytic infiltrate to foci of severe myonecrosis [4].

There is now good evidence that early goal directed therapy (rapid fluid resuscitation and early antibiotics) can save lives in sepsis [5]. However, this requires an early suspicion of superinfection and appreciation of the possibility of less common but more virulent organisms such as MRSA. 

Even though a dengue infection and sepsis were suspected in this patient on admission, there was a low suspicion for MRSA as a causative agent and the patient was not treated with MRSA sensitive antibiotics. The lack of early and aggressive treatment with optimal antibiotics may have been a factor in his clinical deterioration. This patient succumbed to the dual infection and its related complications. We would like to stress that red-coloured urine in dengue patients is not always due to haematuria and if a patient's vital signs do not respond to appropriate fluid management in DHF, sepsis from a secondary pathology including MRSA should be suspected. 

## Figures and Tables

**Table 1:** Investigation results during the illness.

Investigations	2nd day	4th day	5th day
White cell count (mm^−3^)	5300	3200	1600
Neutrophil (%)	67	83	69
Lymphocytes (%)	30	14.7	30
Haemoglobin (g/dL)	13.7	14.9	15.2
Haematocrit	41.80	46.2	46.0
Platelets (mm^−3^)	133000	47000	20000
AST (U/L)		340	1012
ALT (U/L)		85	161
Serum bilirubin (mg/dL)		0.9	1.26
Serum albumin (g/dL)		3.3	2.4
Prothrombin time (s)		13	>60
INR		1.15	>5
APTT			>60
Blood urea (mg/dL)		20	34
Serum creatinine (mg/dL)			1.21
Serum sodium (mmol/L)		131	132
Serum potassium (mmol/L)		4.2	5.3
